# Cost-effectiveness evaluation of quadrivalent influenza vaccines for seasonal influenza prevention: a dynamic modeling study of Canada and the United Kingdom

**DOI:** 10.1186/s12879-015-1193-4

**Published:** 2015-10-27

**Authors:** Edward W. Thommes, Afisi Ismaila, Ayman Chit, Genevieve Meier, Christopher T. Bauch

**Affiliations:** GSK, 7333 Mississauga Road, Mississauga, ON L5N 6L4 Canada; Department of Mathematics & Statistics, University of Guelph, Guelph, Ontario Canada; Department of Clinical Epidemiology and Biostatistics, McMaster University, Hamilton, Ontario Canada; Sanofi Pasteur, Toronto, Ontario Canada; Leslie Dan Faculty of Pharmacy, University of Toronto, Toronto, Ontario Canada; GSK Vaccines, Wavre, Belgium; Department of Applied Mathematics, University of Waterloo, Waterloo, Ontario Canada

**Keywords:** Vaccination, Transmission, Cost-effectiveness, Canada, UK, Influenza, Dynamic, QIV, TIV

## Abstract

**Background:**

The adoption of quadrivalent influenza vaccine (QIV) to replace trivalent influenza vaccine (TIV) in immunization programs is growing worldwide, thus helping to address the problem of influenza B lineage mismatch. However, the price per dose of QIV is higher than that of TIV. In such circumstances, cost-effectiveness analyses provide important and relevant information to inform national health recommendations and implementation decisions. This analysis assessed potential vaccine impacts and cost-effectiveness of a country-wide switch from TIV to QIV, in Canada and the UK, from a third-party payer perspective.

**Methods:**

An age-stratified, dynamic four-strain transmission model which incorporates strain interaction, transmission-rate seasonality and age-specific mixing in the population was used. Model input data were obtained from published literature and online databases. In Canada, we evaluated a switch from TIV to QIV in the entire population. For the UK, we considered two strategies: Children aged 2–17 years who receive the live-attenuated influenza vaccine (LAIV) switch to the quadrivalent formulation (QLAIV), while individuals aged > 18 years switch from TIV to QIV. Two different vaccination uptake scenarios in children (UK1 and UK2, which differ in the vaccine uptake level) were considered. Health and cost outcomes for both vaccination strategies, and the cost-effectiveness of switching from TIV/LAIV to QIV/QLAIV, were estimated from the payer perspective. For Canada and the UK, cost and outcomes were discounted using 5 % and 3.5 % per year, respectively.

**Results:**

Overall, in an average influenza season, our model predicts that a nationwide switch from TIV to QIV would prevent 4.6 % influenza cases, 4.9 % general practitioner (GP) visits, 5.7 % each of emergency room (ER) visits and hospitalizations, and 6.8 % deaths in Canada. In the UK (UK1/UK2), implementing QIV would prevent 1.4 %/1.8 % of influenza cases, 1.6 %/2.0 % each of GP and ER visits, 1.5 %/1.9 % of hospitalizations and 4.3 %/4.9 % of deaths. Discounted incremental cost-utility ratios of $7,961 and £7,989/£7,234 per quality-adjusted life-year (QALY) gained are estimated for Canada and the UK (UK1/UK2), both of which are well within their respective cost-effectiveness threshold values.

**Conclusions:**

Switching from TIV to QIV is expected to be a cost-effective strategy to further reduce the burden of influenza in both countries.

**Electronic supplementary material:**

The online version of this article (doi:10.1186/s12879-015-1193-4) contains supplementary material, which is available to authorized users.

## Background

Influenza A and B viruses are notable respiratory pathogens and remain an important cause of public health concern worldwide [1, 2], with annual influenza attack rates ranging from 5–10 % in adults to 20–30 % in children [1]. While the influenza B virus has often been regarded to be milder than influenza A, several studies have reported that they both cause disease of similar severity, symptoms and rates of influenza-related complications [3–6]. Influenza A and B both cause annual epidemics in individuals of all ages, with B accounting for about a quarter of cases on average, although the proportion can vary substantially from season to season, from less than 1 % to over 50 % [2].

Efficacious and safe influenza vaccines remain the cornerstone of influenza prevention worldwide. Until the 2012–2013 influenza season, only trivalent influenza vaccines were in use, containing two influenza A strains (A/H1N1 and A/H3N2) and only one of the two influenza B lineages, B/Victoria and B/Yamagata. As influenza viruses undergo frequent changes in their surface antigens, the composition of influenza vaccines is changed annually to match the circulating virus subtype expected for the next influenza season, based on the recommendations of the World Health Organization (WHO) [1, 7]. Because there is limited cross-protection between the two influenza B lineages [8, 9], the effectiveness of each season’s trivalent vaccine against influenza B depends on correct prediction of the circulating B lineage [10]. With co-circulation of both influenza B lineages in the last decade [2, 7, 8, 10, 11], this has proven to be challenging; worldwide, the chosen B lineage has been mismatched to the dominant circulating lineage in about half of the seasons. As of the 2013–2014 season, the availability of quadrivalent influenza vaccines, containing both B lineages each season, has offered the potential of improved protection. Indeed, vaccination with quadrivalent vaccine has shown improved immunogenicity, compared with TIV, in children, adults and elderly people [12–15].

Traditionally, annual seasonal influenza vaccination has been targeted to people classified as being at high-risk, particularly the elderly, but in recent years, with increasing evidence in favor of universal vaccination [16], vaccination recommendations have been expanded to target larger numbers and diverse population subgroups. Only some countries now recommend universal influenza immunization. In Canada, publicly-funded universal influenza immunization programs exist in all provinces except British Columbia, Quebec and New Brunswick [17]. The United Kingdom (UK) has until recently had a targeted influenza immunization program with publicly-funded immunization only for people aged 65 years and over, plus the clinical at-risk population. However, beginning with the 2013–2014 season, rollout of a phased extension of the immunization program to healthy children has commenced [18, 19].

Cost-effectiveness analyses are widely used and accepted to explore and understand the impact of different strategies and interventions in diverse settings. Recently performed cost-effectiveness studies using static cohort models show that switching from TIV to quadrivalent influenza vaccine (QIV) in universal programs is a cost-effective strategy [20–23]. Static models cannot however fully model the impact of plausible herd effects which are known to be afforded by vaccination [22, 24]. Moreover, the variability of model outcomes that is attributable to age-specific disease transmission parameters crucial in understanding infectious diseases has not been considered [21]. Dynamic transmission models are the preferred choice when analyzing influenza [25]. The aim of this modeling study using an age-stratified dynamic transmission disease model was to assess the public health and economic impact of a nationwide switch from TIV to QIV, in Canada and the UK, from the perspective of the healthcare provider (third party payer).

## Methods

### Model overview

We used a previously published compartmental (Susceptible-Infected-Recovered-Vaccinated) dynamic transmission model capturing the pairwise interactions of two influenza A strains, A/H1N1 and A/H3N2, and two influenza B lineages, B/Yamagata and B/Victoria [26]. Interactions between the strains were assumed to occur via both natural and vaccine-conferred partial cross-reactive immunity. We assumed that influenza A to B cross-protection is negligible, and modeled only pairwise cross-protection between the two A strains, and between the two B lineages, respectively. The overall structure is thus of a pair of essentially independent two-strain models. Age-dependent contact patterns were specified using a contact matrix [27, 28] to calculate the force of infection. This model was run over a 10-year time horizon, following a 30-year burn-in period. A detailed description of model structure, assumptions and calibration methodology are given in [26]

The model’s structure makes it capable of reproducing the key transmission dynamics of seasonal influenza, specifically herd immunity, strain interaction, waning immunity and dependence on population contact patterns. A small background contribution to the force of infection (corresponding to case importation) varies randomly from season to season, (separately for influenza A and B), thus rendering individual simulations stochastic. Model parameters were fit using an approximately Bayesian computation (ABC) parameter fitting scheme [29], similar to those used previously for fitting human papillomavirus models [30, 31]. The model was calibrated using, as fitting targets, the United States of America (US) unvaccinated (natural) influenza attack rates and year-to-year relative amount of influenza A compared to influenza B [21, 32]. Calibrating on only the unvaccinated population removes direct dependence of the attack rate on the efficacy and uptake of influenza vaccine in the population, though it is still coupling through herd immunity; for this reason the season-by-season vaccine uptake in the US population was also included in the calibration. Further details of the calibration process are given in [26]. The resultant posterior distribution of sets of influenza natural history parameters was then applied to the populations of two countries considered in this analyses - Canada and the UK. The detailed methodology of the calibration of this model is described in Thommes et al. [26].

### Model input data and assumptions

Baseline demographic, cost, utility and vaccine-related input parameters for the dynamic transmission model were obtained from locally available databases and published literature, details of which are described below (for details see Additional file 1).

#### Intervention strategy

In Canada, where TIV is predominantly used in provincial or territorial public programs, we evaluated a full switch from TIV to QIV in individuals of all age groups, at the current nationwide vaccine uptake level (2014). To capture current recommendations and practice in the UK [18, 19], we evaluated two strategies: Children aged 2–17 years who receive the live-attenuated influenza vaccine (LAIV), while individuals of ages 18 and above receive TIV. In this analysis, the latter age group undergoes a switch from TIV to QIV and children aged 2–17 years undergo an analogous switch from the trivalent formulation of LAIV to the quadrivalent formulation of LAIV (QLAIV). Given that the pediatric vaccination program in the UK is currently in its rollout phase, two different vaccination uptake scenarios in children (denoted UK1 and UK2) were evaluated.

#### Demographics

Demographic data, including birth and all-cause mortality rates for Canada were based on the year 2012 and were obtained from the Statistics Canada’s CANSIM online database [33]. For the UK, demographic data, birth and all-cause mortality rates were obtained from the Office of National Statistics (data based on mid-2010 population estimates) [34]. Age-dependent contact patterns specific to Canada are unavailable and hence data from the US were used [35]. For the UK, the relevant contact matrix from Mossong et al. (physical and non-physical contacts) was used [36]. The UK matrix is in terms of number of daily contacts, whereas the US matrix is in terms of daily minutes of contact; since the natural history parameter calibration was performed using the US matrix, the UK matrix had to be converted. We did this assuming a linear relationship between daily minutes and the number of contacts, with the scaling factor chosen to yield the same dominant eigenvalue for UK matrix as for the US.

#### Outcome probabilities

Four outcomes of symptomatic influenza were considered in this analysis – general practitioner (GP) visit, emergency room (ER) visit, hospitalization and death. In the absence of available Canada-specific outcomes probabilities, the US-derived values of Molinari et al. [37] for GP visits, hospitalization and death were used. Probability of an ER visit was derived from the probability of hospitalization using a fixed ratio between the two quantities [24]. For the UK, these outcome probabilities were obtained from Turner et al. [32] (GP visit), Tappenden et al. [38] (ER visit and hospitalization) and Meier et al. [39] (death) (Additional file 1, see Table 1.1, 1.2).

#### Utilities

Age-specific life-expectancy was obtained from the Life Tables and Interim Life Tables for Canada [40, 41] and the UK [42], respectively. Baseline utilities for Canada and the UK were obtained from Mittmann et al. [43] and Tappenden et al.[38]*,* respectively (Additional file 1, see Table 1.3). For Canada, quality-adjusted life-year (QALY) loss per uncomplicated case and medically-attended influenza case were obtained from Tarride et al. [24] and Sander et al. [44], respectively. For the UK, these quantities were obtained from Tappenden et al. [38] (Additional file 1, see Table 1.4).

#### Vaccine uptake

Vaccine uptake rates for Canada in children aged 6–23 months and 2–11 years were obtained from Moran et al. [45] and for individuals aged ≥12 years from Statistics Canada’s CANSIM online database [33] (Additional file 1, see Table 1.5). For the UK, individuals, clinically at-risk (ages 18–64 years and 65+ years) are vaccinated and a universal childhood vaccination program for children of ages 2–17 years is being phased in as of 2013. Given the ongoing changes in child vaccine uptake, we thus considered two scenarios (UK1 and UK2) which differ only in the vaccine uptake in children 2–17 years of age (Additional file 1, see Table 1.6).

#### Vaccine efficacy and adverse events

Vaccine efficacy against influenza A was assumed to be identical for both TIV and QIV. The average efficacy of TIV against influenza A was estimated from three Cochrane reviews in healthy children [46] adults [47] and elderly individuals [48]. This is also supported on the basis of non-inferiority data of QIV and TIV from a vaccine effectiveness study comparing both vaccines [49]. TIV efficacy against influenza B for both a lineage match and mismatch was obtained from a meta-analysis of clinical trials [8]. QIV efficacy against both lineages of influenza B was assumed to always be that of TIV against the matched B lineage. Due to limitations in LAIV influenza B match-mismatch efficacy data in children of ages 3 and above [8], identical efficacies were used for matched LAIV, mismatched LAIV and QLAIV for ages 3–17 years (Additional file 1, see Table 1.7, 1.8). Vaccine-conferred protection against both influenza A and B was assumed to last only one year on average [50].

Data from clinical trials show that QIV and TIV have similar safety profiles [13, 15, 49]. Moreover, with both vaccines the occurrence of adverse events was low, and when present transient in nature [38]. Thus we excluded this parameter in the model.

#### Costs associated with resource use

All costs are reported in the national currencies of the two countries, i.e. the Canadian dollar ($) and Great British Pound (£). The reference year for costs was 2013. For Canada, when costs were unavailable for 2013, they were inflation-adjusted to 2013 using the Canadian Consumer Price Index [33]. In this analysis we considered the payer perspective and therefore only direct medical costs were included. Costs per GP and ER visit for Canada were obtained from Tarride et al. [24] For cost per hospitalization, data from the Canadian Institute for Health Information (CIHI) Patient Cost Estimator tool was used [51]. UK cost data was obtained using multiple sources, analogous to Van Bellinghen et al. [22] (Additional file 1, see Table 1.9).

#### Vaccination costs

For Canada, the blended price of TIV was estimated as an average from published sources at $6.18 per dose [24, 44, 52]. For the UK, the price of TIV was calculated as a weighted average of all TIV available on the UK market and estimated at £6.39 per dose [53]. For QIV in the UK, the list price of £9.94 per dose was used [54]. This constitutes a TIV-QIV price difference of a factor of ~1.56; a hypothetical QIV price for Canada of $9.61 per dose was derived assuming the same relative price difference. Finally, for LAIV and QLAIV, modeled only in the UK scenarios, the list price of £14.00 per dose was used [54]. For Canada, cost of vaccine administration is taken to be at $3.78 [44]. In the UK, vaccination was assumed to take place as part of a regular GP visit, and thus to incur no additional vaccine administration cost (Additional file 1, see Table 1.9).

### Analyses

#### Base case analyses

Health and cost outcome measures resulting from the two vaccination strategies (QIV and TIV) and the difference between QIV and TIV are estimated. For Canada, calculations were performed using a discount rate of 5 % per year for monetary and utility costs as well as outcomes [55]. In the case of the UK, a discount rate of 3.5 % per year was applied to costs and outcomes [56] (see Additional file 1, Table 1.10). To determine the cost-effectiveness of implementing a switch from TIV to QIV, the incremental cost-utility ratio (ICUR) was calculated from the third party payer perspective. Cost-effectiveness thresholds of $40,000–50,000 per QALY gained [44] and £20,000 per QALY gained [57] were assumed for Canada and the UK, respectively.

To simulate the impact of a switch from TIV to QIV in Canada and the UK, we performed for each country a set of 1,000 pairs of simulations with the influenza natural history input parameters for each pair drawn from the aforementioned posterior parameter sets. Each pair consists of one simulation in which a switch from TIV to QIV occurs in the 2014–2015 season (the intervention), and another in which the use of TIV is continued (the comparator). For each simulation, results were recorded from the beginning of the 2014–2015 season to the end of the 2023–2024 season, i.e. over ten seasons. Seasonal averages of influenza infections were estimated for this time period.

#### Deterministic sensitivity analyses

In each scenario evaluated, the distributions of outcomes produced by our ensemble of 1,000 simulation pairs reflects both our uncertainty about the true natural history parameters of influenza, and the stochastic nature of the individual simulations. In other words, our ensemble of simulations constitutes a probabilistic sensitivity analysis (PSA) on natural history parameters of influenza as well as on the variability of influenza seasons.

We also performed a combination of univariate and multivariate sensitivity analyses to investigate the sensitivity of the model outcome, specifically, of the point estimate of cost per QALY gained (i.e. the ICUR) estimated by the model. Different parameters were varied:

- ▪QIV price per dose (Canada and UK) to the upper and lower limits of their respective range values, see Additional file 1, Table 1.9.
- ▪QALY loss (one-sided analysis; for the lower bound, we neglected all non-death QALY loss),
- ▪All breakthrough infection outcomes: If vaccination reduces the severity of influenza in breakthrough infections (i.e. infections of people who are vaccinated within the current season), this has the potential to reduce the benefit of QIV relative to TIV. In this sensitivity analysis (multivariate and one-sided), the most extreme possible scenario was considered, wherein TIV and QIV both have 100 % efficacy against all influenza-associated outcomes (GP visit, ER visit, hospitalization and death). This was done by simultaneously setting all the corresponding outcomes probabilities to zero for breakthrough infections.
- ▪Probability of hospitalization (P_hosp), breakthrough infection: Like all breakthrough infection outcomes above, but with 100 % vaccine efficacy only against hospitalization. Vaccine efficacy against all other outcomes is as in the base case, equal to the vaccine efficacy against infection.
- ▪Outcomes probabilities: Multivariate analysis with all outcome probabilities varied simultaneously at their 95 % confidence interval (CI) lower and upper limits (see Additional file 1, Table 1.1, Table 1.2).
- ▪Discount rate: For Canada (base case = 5.0 %) and the UK (base case = 3.5 %) one-sided sensitivity analysis using 3.5 % and 5.0 % discount rates, respectively, and,
- ▪GP cost, ER cost, and hospitalization cost: For Canada and the UK to the upper and lower limits of their respective range values (see Additional file 1, Table 1.9).

## Results

### Base case analyses

#### Health outcomes

Overall, in an average influenza season, our model predicts that a nationwide switch from TIV to QIV would prevent in Canada, 4.6 % (n = 135,538) of influenza cases, 4.9 % (n = 52,200) GP visits, 5.7 % (n = 3,395) ER visits, 5.7 % (n = 1,876) hospitalizations and 6.8 % (n = 328) deaths (Table 1). In the UK1 scenario (*i.e.* vaccine uptake among children as of 2013), a switch from TIV to QIV is expected to prevent 1.4 % (n = 88,755) influenza cases, 1.6 % (n = 22,917) GP visits, 1.6 % (n = 709) ER visits, 1.5 % (n = 1,050) hospitalizations and 4.3 % (n = 230) deaths. In the UK2 scenario (higher than UK1; projected target vaccine uptake among children in the future), switching from the trivalent to the quadrivalent formulation is expected to have a slightly higher impact than that observed with the UK1 scenario. Under the assumptions of the UK2 scenario, the model predicts that a switch from the trivalent to the quadrivalent formulation would prevent 1.8 % (n = 97,787) influenza cases, 2.0 % (n = 25,028) GP visits, 2.0 % (n = 774) ER visits, 1.9 % (n = 1,154) hospitalizations and 4.9 % (n = 234) deaths (Table 2).

**Table 1:** Overall mean health and cost outcomes for Canada

	TIV	QIV	Difference (TIV vs. QIV)	% Difference
Average influenza-related outcomes per season, n (95 % CI)				
Cases	2,933,460 (2,532,276; 3,351,695)	2,797,922 (2,392,853; 3,199,681)	−135,538 (−228,154; −76,677)	−4.6 (−7.7; −2.7)
GP visits	1,066,568 (921,034; 1,218,892)	1,014,368 (868,298; 1,160,118)	−52,200 (−88,460; −29,055)	−4.9 (−8.2; −2.8)
ER visits	59,704 (51,257; 68,574)	56,309 (47,987; 64,721)	−3,395 (−5,907; −1,731)	−5.7 (−9.7; −3.0)
Hospitalizations	32,986 (28,319; 37,886)	31,110 (26,512; 35,757)	−1,876 (−3,264; −956)	−5.7 (−9.7; −3.0)
Deaths	4,836 (4,114; 5,606)	4,508 (3,811; 5,230)	−328 (−584; −156)	−6.8 (−11.9; −3.2)
Average costs per season, $ (95 % CI)				
Vaccination	$114,269,815 (113,818,022; 114,795,606)	$153,621,770 (153,014,388; 154,328,631)	$39,351,954 (39,196,367; 39,533,025)	
GP visits	$45,574,462 (39,355,763; 52,083,266)	$43,343,948 (37,102,387; 49,571,823)	-$2,230,514 (−3,779,889; −1,241,510)	
ER visits	$13,337,288 (11,450,298; 15,318,666)	$12,578,851 (10,719,802; 14,457,926)	-$758,437 (−1,319,645; −386,684)	
Hospitalization	$120,689,432 (103,441,040; 138,855,265)	$113,695,532 (96,748,864; 130,839,986)	-$6,993,900 (−12,204,664; −3,525,203)	
Total payer costs	$293,870,997 (268,805,179; 320,376,234)	$323,240,101 (298,328,487; 348,108,942)	$29,369,104 (22,016,160; 34,131,5416)	
Total costs, 2014–24: 5 % discounted, $ (95 % CI)				
Vaccination	$868,017,693 (864,552,580; 872,000,633)	$1,166,943,465 (1,162,285,045; 1,172,298,039)	$298,925,772 (297,732,465; 300,297,407)	
GP visits	$347,201,538 (298,121,148; 398,164,181)	$329,329,994 (280,856,904; 378,138,361)	$-17,871,544 (−29,416,959; −9,870,111)	
ER visits	$101,255,332 (86,543,184; 116,554,469)	$95,274,727 (80,828,208; 109,823,373)	$-5,980,605 (−10,120,952; −3,025,115)	
Hospitalization	$915,691,926 (781,948,765; 1,054,610,228)	$860,655,203 (728,830,865; 993,481,676)	$-55,036,723 (−93,315,528; −27,516,515)	
Total payer costs	$2,232,166,489 (2,035,062,286; 2,436,875,507)	$2,452,203,389 (2,261,195,912; 2,649,106,709)	$220,036,899 (166,524,687; 257,670,736)	
QALYs lost, n (95 % CI)				
Per season	68,980 (59,036; 79,436)	64,930 (55,206; 74,837)	−4,050 (−7,076; −2,033)	
Total	522,596 (446,330; 601,554)	490,805 (414,820; 567,537)	−31,791 (−54,079; −15,845)	
LYs lost, n (95 % CI)				
Per season	45,675 (38,909; 52,852)	42,732 (36,152; 49,573)	−2,944 (−5,215; −1,417)	
Total	344,912 (293,245; 398,169)	322,013 (270,885; 374,069)	−22,899 (−39,878; −10,871)	

Note: A negative value for the difference denotes outcomes prevented; GP, general practitioner; ER, emergency room; QALYs, quality-adjusted life years; LYs, life years

**Table 2:** Overall mean health and cost outcomes for the UK

UK1				
	TIV/LAIV	QIV/QLAIV	Difference (TIV/LAIV vs QIV/QLAIV)	% Difference
Average influenza-related outcomes per season, n (95 % CI)				
Cases	6,175,473 (5,348,157; 7,095,022)	6,086,718 (5,244,706; 6,981,581)	−88,755 (−153,607; −46,761)	−1.4 (−2.5; −0.8)
GP visits	1,477,243 (1,276,226; 1,698,398)	1,454,327 (1,249,078; 1,672,087)	−22,917 (−39,758; −12,127)	−1.6 (−2.7; −0.8)
ER visits	45,688 (39,471; 52,528)	44,979 (38,631; 51,714)	−709 (−1,230; −375)	−1.6 (−2.7; −0.8)
Hospitalizations	71,740 (62,217; 82,455)	70,690 (61,026; 81,103)	−1,050 (−1,824; −553)	−1.5 (−2.5; −0.8)
Deaths	5,366 (4,609; 6,207)	5,136 (4,372; 5,941)	−230 (−424; −107)	−4.3 (−7.5; −2.0)
Average costs per season, £ (95 % CI)				
Vaccination	£142,869,897 (142,132,194; 143,838,565)	£173,690,646 (172,847,546; 174,801,973)	£30,820,749 (30,714,458; 30,964,151)	
GP visits	£54,658,003 (47,220,348; 62,840,730)	£53,810,083 (46,215,893; 61,867,225)	-£847,920 (−1,471,032; −448,693)	
ER visits	£6,167,872 (5,328,571; 7,091,250)	£6,072,188 (5,215,223; 6,981,395)	-£95,683 (−165,998; −50,633)	
Hospitalization	£316,143,107 (273,230,094; 363,500,069)	£310,054,168 (266,192,382; 356,545,209)	-£6,088,939( −10,684,477; −3,156,736)	
Total payer costs	£519,838,878 (469,625,943; 576,456,567)	£543,627,085 (491,853,288; 598,787,408)	£23,788,206 (18,451,500; 27,182,411)	
Total costs, 2014–24: 3.5 % discount, £ (95 % CI)				
Vaccination	£1,182,629,311 (1,176,522,193; 1,190,629,232)	£1,438,387,119 (1,431,392,398; 1,447,571,973)	£255,757,808 (254,873,033; 256,946,194)	
GP visits	£449,966,551 (390,148,320; 516,657,013)	£442,621,741 (381,366,507; 508,088,148)	-£7,344,811 (−12,406,117; −3,925,542)	
ER visits	£50,776,387 (44,026,211; 58,302,059)	£49,947,563 (43,035,229; 57,335,107)	-£828,824 (−1,399,966; −442,977)	
Hospitalization	£2,603,109,409 (2,257,958,720; 2,989,825,442)	£2,550,795,544 (2,197,010,408; 2,929,281,052)	-£52,313,865 (−89,406,033; −26,864,867)	
Total payer costs	£4,286,481,659 (3,871,840,486; 4,753,225,888)	£4,481,751,967 (4,063,534,469; 4,933,835,213)	£195,270,308 (152,116,034; 224,188,224)	
QALYs lost, n (95 % CI)				
Per season	138,681 (119,774; 159,443)	135,415 (116,128; 155,866)	−3,266 (−5,749; −1,616)	
Total	1,143,215 (990,661; 1,314,381)	1,115,337 (958,023; 1,283,316)	−27,878 (−48,670; −13,753)	
LYs lost, n (95 % CI)				
Per season	67,761 (58,243; 78,145)	65,340 (55,700; 75,446)	−2,421 (−4,407; −1,150)	
Total	560,869 (482,171; 647,615)	540,396 (459,999; 623,573)	−20,473 (−37,239; −9,741)	
UK2				
	TIV/LAIV	QIV/QLAIV	Difference (TIV/LAIV vs QIV/QLAIV)	% Difference
Average influenza-related outcomes per season, n (95 % CI)				
Cases	5,335,431 (4,663,367; 6,094,108)	5,237,644 (4,563,870; 5,976,162)	−97,787 (−167,789; −53,233)	−1.8 (−3.0; −1.0)
GP visits	1,279,954 (1,116,137; 1,463,587)	1,254,916 (1,091,268; 1,432,823)	−25,038 (−42,527; −13,756)	−2.0 (−3.2; −1.1)
ER visits	39,586 (34,520; 45,266)	38,812 (33,751; 44,314)	−774 (−1,315; −425)	−2.0 (−3.2; −1.1)
Hospitalizations	61,812 (54,111; 70,575)	60,658 (52,902; 69,153)	−1,154 (−1,992; −626)	−1.9 (−3.1; −1.1)
Deaths	4,772 (4,146; 5,483)	4,538 (3,921; 5,202)	−234 (−410; −116)	−4.9 (−8.3; −2.5)
Average costs per season, £ (95 % CI)				
Vaccination	£171,724,730 (170,737,027; 172,880,105)	£202,522,845 (201,431,528; 203,801,765)	£30,798,116 (30,696,168; 30,920,847)	
GP visits	£47,358,314 (41,297,066; 54,152,709)	£46,431,902 (40,376,915; 53,014,460)	-£926,412 (−1,573,508; −508,968)	
ER visits	£5,344,140 (4,660,159; 6,110,852)	£5,239,599 (4,556,325; 5,982,406)	-£104,541 (−177,562; −57,434)	
Hospitalization	£274,233,102 (239,080,248; 313,959,886)	£267,724,197 (232,618,317; 305,657,406)	-£6,508,906 (−11,183,958; −3,502,201)	
Total payer costs	£498,660,286 (457,399,436; 545,711,073)	£521,918,543 (480,857,526; 567,317,462)	£23,258,257 (17,987,595; 26,732,485)	
Total costs, 2014–24, 3.5 % discount, £ (95 % CI)				
Vaccination	£1,421085,767 (1,412,898,941; 1,430,629,196)	£1,676,655,043 (1,667,605,341; 1,687,226,238)	£255,569,276 (254,720,544; 256,584,332)	
GP visits	£390,019,191 (338,972,997; 445,806,414)	£382,011,087 (331,356,732; 436,393,837)	-£8,008,104 (−13,328,069; −4,445,418)	
ER visits	£44,011,639 (38,251,341; 50,306,937)	£43,107,966 (37,391,885; 49,244,777)	-£903,673 (−1,504,003; −501,642)	
Hospitalization	£2,258,890,346 (1,965,081,255; 2,581,768,165)	£2,203,039,151 (1,911,631,217; 2,513,877,596)	-£55,851,195 (−94,174,503; 30,373,049)	
Total payer costs	£4,114,006,943 (3,771,593,455; 4,497,105,857)	£4,304,813,247 (3,962,279,878; 4,678,534,060)	£190,806,304 (147,019,450; 220,067,782)	
QALYs lost, n (95 % CI)				
Per season	121,040 (105,258; 138,570)	117,603 (102,122; 134,418)	−3,437 (−5,970; −1,802)	
Total	998,215 (867,812; 1,140,983)	968,900 (839,411; 1,106,335)	−29,315 (−49,909; −15,593)	
LYs lost, n (95 % CI)				
Per season	59,931 (51,974; 68,794)	57,448 (49,677; 65,930)	−2,484 (−4,309; −1,247)	
Total	496,291 (429,909; 568,923)	475,295 (409,468; 545,073)	−20,996 (−36,237; −10,647)	

Note: A negative value for the difference denotes outcomes prevented; GP, general practitioner; ER, emergency room; QALYs, quality-adjusted life years; LYs, life years

Outcomes results are also reported in age-stratified form for age groups 0–4, 5–19, 20–49, 50–64, 65–74, 75–84 and 85–99 years; see Additional file 2, Additional file 3. This presentation provides additional insight. For example, in Canada the percentage of deaths prevented across all ages is higher than that of cases prevented, and in the UK the difference is even more pronounced. Now the case fatality ratio for influenza i.e. probability of death due to influenza rises sharply in individuals of ages above 65 years in both countries (Additional file 1, Table 1.1 and Table 1.2), thus the percentage of overall deaths prevented depends most strongly on the percentage of cases prevented in the elderly. Indeed, in both Canada and the UK scenarios, the percentage of cases prevented increases with age similar to vaccine uptake. This features more strongly in the UK where healthy individuals aged 18–64 years are not part of the public vaccination program.

**Fig. 1:**
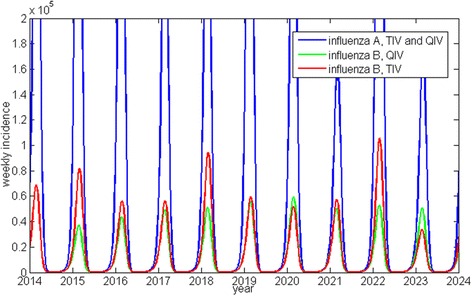
Comparison of a typical TIV-QIV pair of model simulations (example of Canada). Note: The seventh and tenth influenza B peaks are actually slightly higher with QIV than with TIV. This is because the model is stochastic; TIV and QIV versions diverge so there is no real 1:1 correspondence between seasons after the first season in which QIV is introduced; Since TIV and QIV have identical efficacy against influenza A, the evolution of influenza A incidence with the TIV and QIV is identical between the pair of simulations

**Fig. 2:**
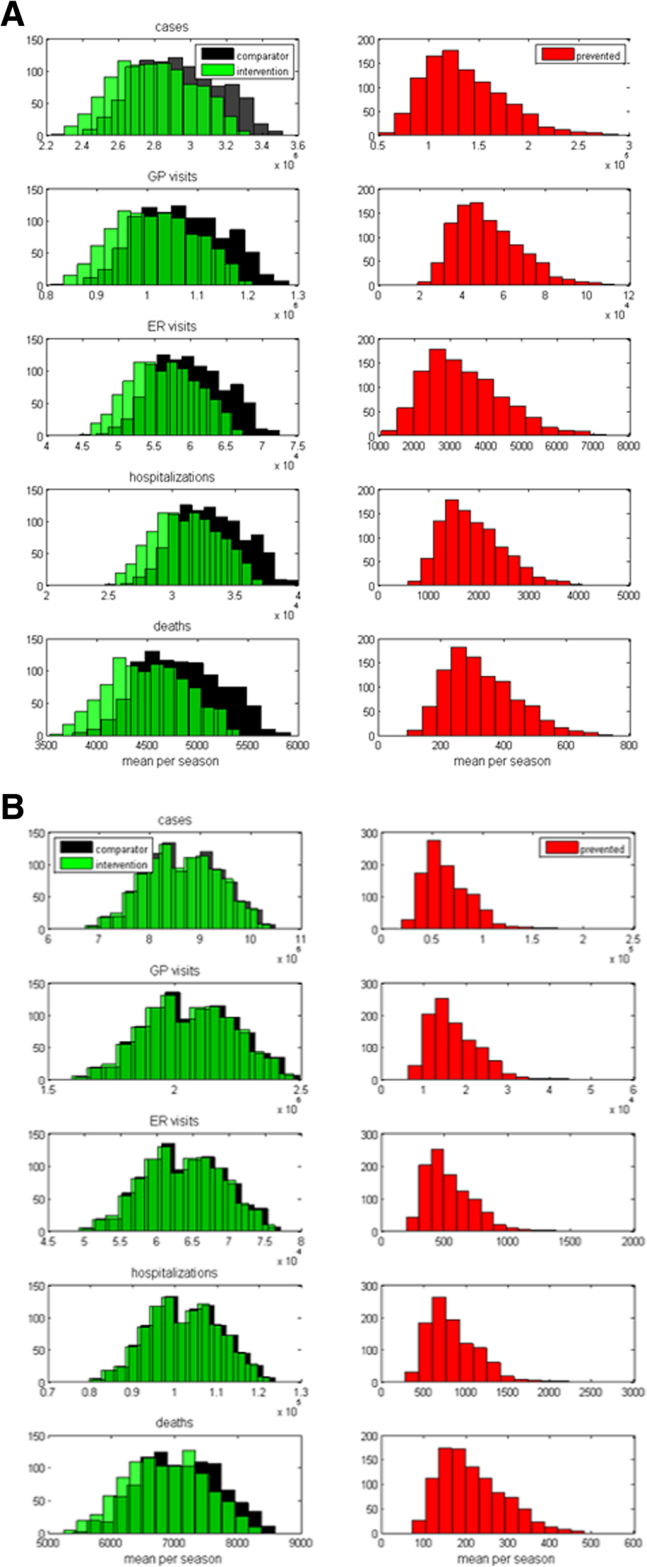
Distribution of mean influenza-related outcomes per season (**a**) Canada (**b**) UK. Note: Outcomes are presented for TIV (comparator) and QIV (intervention) and mean outcomes prevented resulting from a switch from TIV to QIV (prevented)

A typical example of one pair of Canadian simulations, showing the impact of switching from TIV to QIV, is shown in Fig. 1. The distribution of model results for mean outcomes per season across all age groups with TIV, QIV and the difference between the two interventions per simulation pair, for Canada and the UK (1,000 model results each), are shown in Fig. 2.

#### Cost-effectiveness analysis

In Canada, discounting at 5 %, a switch from TIV to QIV would result in costs of $224, $588, $9,407, $17,206 and $10,1215 per influenza case, GP visit, ER visit, hospitalization and death averted, respectively. Costs per QALY and life-year (LY) gained were estimated as $7,961 (95 % CI: $3,080–$16,320) and $11,211 (95 % CI: $4,181–$23,815), respectively (Table 3).

**Table 3:** Modeled ICURs for Canada (discounted, 5 %)

Category	Mean value (95 % CI)
Cost per case averted	$224 (94; 419)
Cost per GP visit averted	$588 (243; 1120)
Cost per ER visit averted	$9,407 (3,678; 19,086)
Cost per hospitalization averted	$17,026 (6,658; 34,546)
Cost per death averted	$101,215 (37,429; 216,888)
Cost per QALY gained	$7,961 (3,080; 16,320)
Cost per LY gained	$11,211 (4,181; 23,815)

Note: GP, general practitioner; ER, emergency room; QALY, quality-adjusted life year; LY, life year

For the UK (scenarios UK1/UK2), discounting at 3.5 %, a switch from TIV to QIV would result in costs of £282/£250, £1,100/£979, £35,568/£31,641, £23,929/£21,207, and £117,428/£110,496 per influenza case, GP visit, ER visit, hospitalization and death averted, respectively. Discounted costs per QALY and LY gained were estimated at £7,989 (95 % CI: 3,132–16,318)/£7,324 (95 % CI: 2,937–14,044) and £11,081 (95 % CI: 4,135–23,186)/£10,364 (95 % CI: 4,029–20,588), respectively (Table 4).

**Table 4:** Modeled ICURs for the UK (discounted, 3.5 %)

Outcomes, 3.5 % discount	Mean value (95 % CI)
UK1	
Cost per case averted	£282 (118; 538)
Cost per GP visit averted	£1,100 (456; 2,113)
Cost per ER visit averted	£35,568 (14,729; 68,327)
Cost per hospitalization averted	£23,929 (9,934; 45,494)
Cost per death averted	£117,428 (43,067; 246,342)
Cost per QALY gained	£7,989 (3,132; 16,318)
Cost per LY gained	£11,081 (4,135; 23,186)
UK2	
Cost per case averted	£250 (105; 461)
Cost per GP visit averted	£979 (404; 1,821)
Cost per ER visit averted	£31,641 (13,069; 58,892)
Cost per hospitalization averted	£21,207 (8,909; 39,220)
Cost per death averted	£110,496 (42,719; 221,654)
Cost per QALY gained	£7,324 (2,937; 14,044)
Cost per LY gained	£10,364 (4,029; 20,588)

Note: GP, general practitioner; ER, emergency room; QALY, quality-adjusted life year; LY, life year

**Fig. 3:**
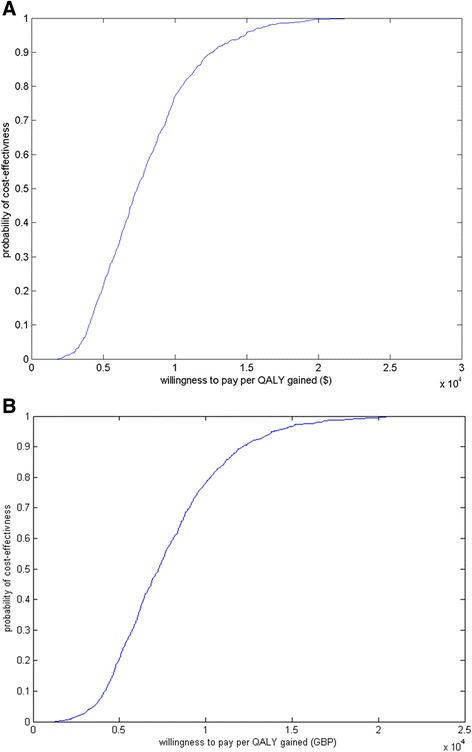
Cost-effectiveness acceptability curves (**a**) Canada (**b**) UK^‡^. Note: QALY, quality-adjusted life-year; Cost-effectiveness threshold for Canada and UK are $40,000–50,000 and £20,000 per QALY gained, respectively; ^‡^Figure presented for the UK is based on Scenario UK1, UK2 is very similar (data not shown)

The model results for the ICUR are presented as a cost-effectiveness acceptability curve, which shows, as a function of the adopted cost-effectiveness threshold, the probability that a switch from TIV to QIV would be cost-effective. For Canada, considering a threshold of $40,000–50,000 per QALY gained [44], a switch from TIV to QIV is predicted to have a 100 % probability of cost-effectiveness. For the UK, considering a threshold of £20,000 per QALY gained [57], the model predicts a probability of cost-effectiveness greater than 99 % (Fig. 3).

### Deterministic sensitivity analyses

**Fig. 4:**
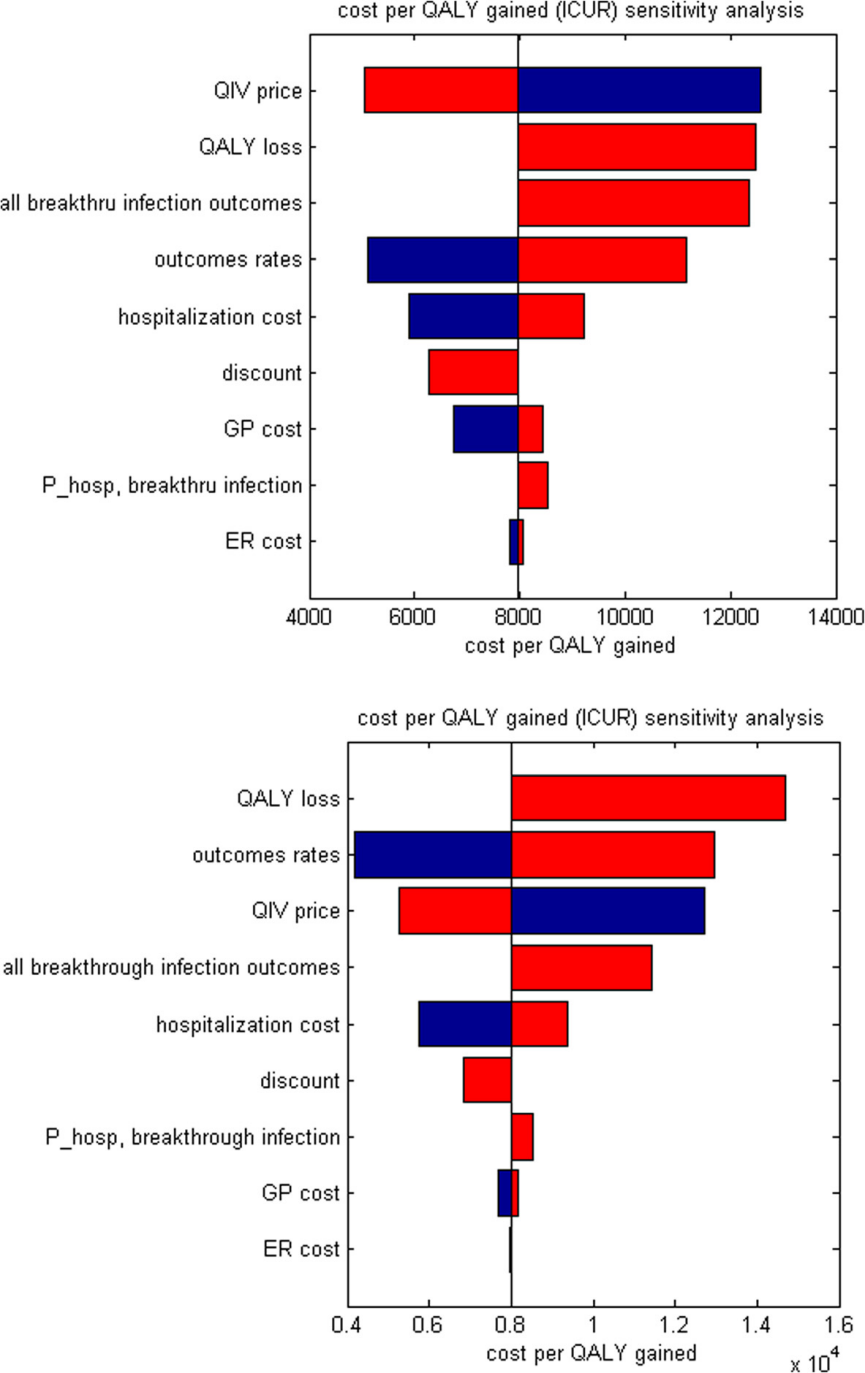
Deterministic sensitivity analyses (**a**) Canada (**b**) UK^‡^. Note: The grey vertical line corresponds to all the uncertain parameters being at their respective base values. The red segments of the bars correspond to result values increasing the base case ICUR and the blue segments of the bars correspond to result values decreasing the base case ICUR; ^‡^Figure presented for the UK is based on Scenario UK1, UK2 is very similar (data not shown); ER, emergency room; GP, general practitioner, QIV, quadrivalent influenza vaccine; QALY, quality-adjusted life-year

Deterministic sensitivity analyses for both the countries indicate that among the model parameters varied, the modelled ICUR is most sensitive to the chosen range of QIV price per dose, outcome probabilities (varied simultaneously), to whether QALY loss due to non-fatal influenza is taken into account, and to a hypothetical extreme case scenario in which breakthrough infections never result in a GP visit, ER visit, hospitalization or death, regardless of the type of vaccine used. For Canada, the ICUR varies from a minimum of $5,000 per QALY (lower QIV price) to a maximum of $12,600 per QALY (higher QIV price), while for the UK it varies from a minimum of £4,000 per QALY (high values for all outcomes rates) to a maximum of £14,200 per QALY (QALY loss due to non-fatal influenza neglected) (Fig. 4). Comparison to the accepted cost-effectiveness thresholds of $40,000–$50,000 per QALY gained and £20,000 per QALY gained in Canada and the UK respectively, suggests a high likelihood of cost-effectiveness in both countries, especially Canada, notwithstanding uncertainty in rates of outcomes and costs.

## Discussion

This analysis aimed to assess the potential impact of switching from TIV to QIV over ten seasons in Canada and the UK. The dynamic transmission model in this analysis provides a detailed, age- and strain or lineage-stratified representation of influenza dynamics, calibrated to surveillance data.

The model estimates that implementing a switch from TIV to QIV in both Canada and the UK would result in moderate reductions in the number of influenza cases and influenza-associated outcomes (GP and ER visits, hospitalizations and deaths). Though both countries have broadly similar demographic characteristics, we observed a significantly greater relative impact from the switch in Canada compared to the UK. The difference stems principally from vaccine uptake: While most Canadian provinces have universal influenza immunization programs (free vaccination for ages 6 months and above), the targeted vaccination program in the UK, although currently expanding to cover children, still leaves healthy adults aged 18–64 uncovered, resulting in significantly lower uptake in that age group as compared to Canada [33, 45].

Despite a net increase in payer cost associated with the switch from TIV to QIV at the assumed QIV prices, the use of QIV is projected to be highly cost-effective in both countries, with an ICUR point estimate of $7,961, £7,989 and £7,324 per QALY gained in Canada and in the two UK scenarios, respectively, far below each country’s cost-effectiveness threshold. This conclusion is shown to be robust against uncertainty in the natural history parameters of influenza (as expressed in the cost-effectiveness acceptability curves, Fig. 4) in both settings, albeit more strongly so in Canada. Separate deterministic sensitivity analysis suggests it is also robust against uncertainty in costs and outcomes rates, again specifically in Canada.

A switch from TIV to QIV may carry additional benefits beyond those modeled here. Even though we have conservatively assumed that all vaccinated immunity only lasts one year on average, the slower antigenic drift of influenza B may result in some carry-over of influenza B protection to subsequent seasons, thus further increasing the benefit of QIV relative to TIV [2]. Also, since vaccination with QIV is expected to result in fewer mismatched seasonal vaccine campaigns, its adoption may result in improved public perception of influenza vaccination, in turn translating into higher vaccine uptake [2].

Only a handful of studies have so far modeled the impact of switching from TIV to QIV on a country scale, in the US [18, 19] and the UK [20, 21, 23]. These studies have reported broadly similar findings to ours, of cost-effectiveness across a broad range of QIV prices. However to our knowledge, there are thus far no published TIV-QIV studies utilizing a dynamic model, and none at all for Canada. Canadian studies of a different influenza vaccine intervention do exist as a basis for comparison, namely the transition in 2000 of the province of Ontario from targeted to universal immunization. A health economic study by Sander et al. [44], utilizing the results of an ecological study by Kwong et al. [58] (which served as a test case for the model we use here [26]), estimated the cost-effectiveness of Ontario’s transition at $12,154 per QALY gained. Our work here thus suggests that the cost-effectiveness of a TIV-QIV switch is comparable to that of a targeted-universal switch.

Our model was chosen for its ability to reproduce key elements of influenza dynamics: herd immunity, waning immunity, seasonality, age-specific contact patterns, and, critically for the problem studied here, the interplay of multiple influenza strains and influenza B lineages. However, like any model, it had limitations. Stratification of the population was only by age; healthy and at-risk parts of the population were not separately tracked. As described in [26], calibration of the natural history parameters of influenza was performed using the US as a setting, thus making the assumption that these differ negligibly between the US, Canada and the UK. It should be emphasized that the distribution of outcomes obtained through the calibration procedure (effectively a probabilistic sensitivity analysis on influenza natural history parameters) does *not* encapsulate uncertainty about the comparability of US to Canadian and UK influenza natural history. Due to the absence of Canadian data, US outcomes probabilities were used for Canada. Limitations in available LAIV influenza B match/mismatch efficacy data caused the LAIV and QLAIV modelled in the UK scenarios differ little in efficacy. Finally, we did not consider adverse events due to vaccination in this analysis. But given that both vaccines have similar safety profiles, this assumption is justified as it would not impact the model result.

## Conclusions

Our findings predict that a switch from TIV to QIV is a highly cost-effective intervention to reduce the burden of influenza in both Canada and the UK, and thus suggest such a switch as a public health policy priority in both settings.

## Additional files

Additional file 1:
**Model input data for Canada and UK.** (DOCX 53 kb) Additional file 2:
**Age-specific mean health outcomes per season in Canada.** (DOCX 51 kb) Additional file 3:
**Age-specific mean health outcomes per season in the UK.** (DOCX 65 kb)

## Abbreviations

CI: confidence interval
CIHI: Canadian Institute for Health Information
ER: emergency room
GP: general practitioner
LAIV: live-attenuated influenza vaccine (trivalent)
ICUR: incremental cost-utility ratio
LY: life-year
PSA: Probabilistic sensitivity analysis
QALY: quality-adjusted life-year
QIV: quadrivalent influenza vaccine
QLAIV: quadrivalent LAIV
TIV: trivalent influenza vaccine
UK: United Kingdom
US: United States of America
WHO: World Health Organization
