# Supplementary material for: Cost-effectiveness evaluation of quadrivalent influenza vaccines for seasonal influenza prevention: a dynamic modeling study of Canada and the United Kingdom
Source: BMC Infect Dis. 2015 Oct 27;15:465. doi: 10.1186/s12879-015-1193-4 (PMC4623926; doi:10.1186/s12879-015-1193-4)
Supplement: Additional file 1: — Model input data for Canada and UK. (DOCX 53 kb) [file 12879_2015_1193_MOESM1_ESM.docx]

1. **Model input data for Canada and UK**
2. **Outcome probabilities for Canada**

| **Probability (P) of outcome resulting from influenza B infection** | **Age (years)** | **Base case value (95%CI)** |
| --- | --- | --- |
| P (GP\|flu) [37] | 0–4 | 0.45500 (0.29302; 0.67519) |
|  | 5–17 | 0.31800 (0.21517; 0.45330) |
|  | 18–49 | 0.31300 (0.28646; 0.34132) |
|  | 50–64 | 0.31300 (0.28646; 0.34132) |
|  | 65+ | 0.62000 (0.56876; 0.67458) |
| P (hosp\|flu) [37] | 0–4 | 0.01410 (0.00708; 0.02527) |
|  | 5–17 | 0.00060 (0.00030; 0.00108) |
|  | 18–49 | 0.00420 (0.00211; 0.00753) |
|  | 50–64 | 0.01930 (0.00973; 0.03450) |
|  | 65+ | 0.04210 (0.02118; 0.07537) |
| P (death\|flu) [37] | 0–4 | 0.00004 (0.00002; 0.00006) |
|  | 5–17 | 0.00001 (0.00001; 0.00001) |
|  | 18–49 | 0.00009 (0.00005; 0.00016) |
|  | 50–64 | 0.00134 (0.00067; 0.00241) |
|  | 65+ | 0.01170 (0.00588; 0.02097) |
| P (ER\|flu) [24] | 0–4 | 0.02552 (0.01755; 0.03590) |
|  | 5–17 | 0.00109 (0.00075; 0.00153) |
|  | 18–49 | 0.00760 (0.00523; 0.01069) |
|  | 50–64 | 0.03493 (0.02407; 0.04906) |
|  | 65+ | 0.07620 (0.05244; 0.10711) |

Note: SA ranges are the 95% CI bounds, assuming beta distributions with standard error half the base [59]; ER, emergency room; GP, general practitioner; hosp, hospitalization

1. **Outcomes probabilities for the UK**

|  | **Age (years)** | **Base case value (95%CI)** |
| --- | --- | --- |
| P (GP\|flu) [32] | 0–12 | 0.1551 (0.0377; 0.3343) |
|  | 13–64 | 0.2819 (0.0593; 0.5930) |
|  | 65+ | 0.3251 (0.0640; 0.6753) |
| P (hosp\|flu) [32] | 0–14 | 0.0154 (0.0042; 0.0337) |
|  | 15–64 | 0.0088 (0.0024; 0.0192) |
|  | 65+ | 0.0144 (0.0039; 0.0315) |
| P (death\|flu) [39] | 0–14 | 0.0000 (0.0000; 0.0000) |
|  | 15–49 | 0.0003 (8.17e-05; 0.0007) |
|  | 50–64 | 0.0007 (0.0002; 0.0015) |
|  | 65+ | 0.0110 (0.0030; 0.0241) |
| P (ER\|flu) [38] | 0–12 | 0.0048 (0.0013; 0.0105) |
|  | 13–64 | 0.00872 (0.0023; 0.0191) |
|  | 65+ | 0.01005 (0.0027; 0.0220) |

Note: SA ranges are the 95% CI bounds, assuming beta distributions with standard error half the base [59]; ER, emergency room; GP, general practitioner; hosp, hospitalization

1. **Baseline age-specific health utilities for Canada and the UK**

| **Canada [43]** | | **UK [38]** | |
| --- | --- | --- | --- |
| **Age (years)** | **Value** | **Age (years)** | **Value** |
| 0–<40 | 0.94 | 0–24 | 0.94 |
| 40–49 | 0.93 | 25–34 | 0.93 |
| 50–59 | 0.92 | 35–44 | 0.91 |
| 60–79 | 0.91 | 45–54 | 0.85 |
| 80–99 | 0.88 | 55–64 | 0.80 |
| - | - | 65–74 | 0.78 |
| - | - | 75+ | 0.73 |

1. **Disutilities for Canada and the UK**

| **Parameter** |  | | |
| --- | --- | --- | --- |
|  | **Canada** | | |
| QALY loss per uncomplicated influenza case [44] | Overall population: 0.0041^‡^ | | |
| QALY loss per medically-attended influenza case (requiring GP visit, ER visit or hospitalization) [44] | **0–19 years** | **20–64 years** | **65+years** |
|  | 0.0146 | 0.0174 | 0.0293 |
|  | **UK** | | |
| QALY loss averaged across all influenza outcomes [38]^†^ | **0–14 years** | **15–64 years** | **65+years** |
|  | 0.0105 | 0.0105 | 0.0205 |

Note: QALY, quality-adjusted life-year

^‡^Average number of symptom days=4.03, Utility loss due to influenza=0.442

^†^An average computed from disutilities, durations and relative probabilities of different outcomes

1. **Vaccine uptake rates in Canada** **[33, 45]**

| Age (years) | 0 | 1 | 2–11 | 12–19 | 20–34 | 35–44 | 45–64 | 65+ |
| --- | --- | --- | --- | --- | --- | --- | --- | --- |
| Vaccine uptake (%) | 16.75 | 32.85 | 28.30 | 22.90 | 16.10 | 20.70 | 31.40 | 64.40 |

1. **Vaccine uptake rates in the UK [60]**

| Age (years) | 0–1 | 2–17 | 18–49 | 50–64 | 65+ |
| --- | --- | --- | --- | --- | --- |
| Vaccine uptake (%) | 0 | UK1 scenario: 52.50 [18] | 3.88^†^ | 17.63^‡^ | 71.13 |
|  | - | UK2 scenario: 70.00 (assumption) | - | - | - |

Note: LAIV, live-attenuated influenza vaccine

^†^only clinical at-risk individuals vaccinate; 11.38% of population, uptake 34.07%

^‡^only clinical at-risk individuals vaccinate; 17.63% of population, uptake 100.00%

1. **Efficacy rates against influenza A** **[46-48]**

| TIV & QIV | | | |
| --- | --- | --- | --- |
| Age (years) | 0–17 | 18–64 | 65+ |
| Vaccine efficacy (%) | 59.00 | 61.00 | 58.00 |
| LAIV & QLAIV (UK) | | | |
| Age (years) | 0–2 | 3–17 | - |
| Vaccine efficacy (%) | 81.00 | 66.00 | - |

Note: TIV, trivalent influenza vaccine; LAIV, live-attenuated influenza vaccine (trivalent); QIV, quadrivalent influenza vaccine; QLAIV, quadrivalent LAIV

1. **Efficacy rates against influenza B** **[8]**

| TIV & QIV |  |  |  |  |  |
| --- | --- | --- | --- | --- | --- |
| Age (years) | **<5** | **5–49** | **50–64** | **65–74** | **75+** |
| TIV efficacy, influenza B lineage match (%) | 66.00 | 77.00 | 73.00 | 69.00 | 66.00 |
| TIV efficacy, influenza B lineage mismatch (%) | 44.00 | 52.00 | 49.00 | 47.00 | 44.00 |
| QIV efficacy | 66.00 | 77.00 | 73.00 | 69.00 | 66.00 |
| LAIV & QLAIV (UK only) |  |  |  |  |  |
| Age (years) | **0–2** | **3–17** | - | - | - |
| LAIV efficacy, influenza B lineage match (%) | 73.00 | 53.00 | - | - | - |
| LAIV efficacy, influenza B lineage mismatch (%) | 34.00 | 53.00 | - | - | - |
| QLAIV efficacy (%) | 73.00 | 53.00 | - | - | - |

Note: TIV, trivalent influenza vaccine; LAIV, live-attenuated influenza vaccine (trivalent); QIV, quadrivalent influenza vaccine; QLAIV, quadrivalent LAIV

1. **Influenza vaccination and treatment costs for Canada and the UK**

| **Parameter** | **Base case value (range)** | |
| --- | --- | --- |
|  | **Canada^‡^** | **UK^†^** |
| TIV price per dose | $6.18 [24,44, 52] | £6.39 [53] |
| QIV price per dose | $9.61 ($8.65, $11.12)[assumption] | £9.94 (£9.00; £11.57) [54] |
| LAIV, QLAIV price per dose | Not applicable | £14.00 [54] |
| Vaccine administration cost per dose | $3.78 [24] | 0 (Vaccination assumed to occur as part of regular GP visit) |
| Cost per GP visit | $42.73 ($6.11; $133.49) [24] | £37.00 (£10.08; £81.10) |
| Cost per ER visit | $223.39 ($103.81; $397.64) [24] | £135.00 (£36.78; £295.90) (weighted over all outpatient procedures) |
| Cost per hospitalization | CIHI Patient Cost Estimator [51] | Cost of Illness Associated With Influenza in the UK [61] |
|  | 0 years: $3,145 ($857; $6,893) | 0–4 years: £2,365 (£645; £5,185) |
|  | 1–7 years: $2,715 ($740; $5,951) | 5–17 years: £3,368 (£918; £7,383) |
|  | 8–17 years: $3,249 ($885; $7,121) | 18–49 years: £5,017 (£1,367; £10,998) |
|  | 18–59 years: $3,522($960; $7,720) | 50–64 years: £7,598 (£2,070; £16,653) |
|  | 60–79 years: $3,781 ($1,030; $8,287) | 65+years: £10,250 (£2,793; £22,465) |
|  | 80+years: $4,498 ($1,226; $9,859) |  |

Note: GP, general practitioner; ER, emergency room; TIV, trivalent influenza vaccine; LAIV, live-attenuated influenza vaccine (trivalent); QIV, quadrivalent influenza vaccine; QLAIV, quadrivalent LAIV

^†^All UK costs are 2013 values; ^‡^ Where needed, Canadian costs have been adjusted to 2013 using the Canadian Consumer Price Index [33]

1. **Discount rates for Canada and the UK**

| **Discount rate for costs and outcomes** | **Canada** | **UK** |
| --- | --- | --- |
| **Base case value** | 5.0% [55] | 3.5% [56] |
