# Supplementary material for: Cost-effectiveness evaluation of quadrivalent influenza vaccines for seasonal influenza prevention: a dynamic modeling study of Canada and the United Kingdom
Source: BMC Infect Dis. 2015 Oct 27;15:465. doi: 10.1186/s12879-015-1193-4 (PMC4623926; doi:10.1186/s12879-015-1193-4)
Supplement: Additional file 2: — Age-specific mean health outcomes per season in Canada. (DOCX 51 kb) [file 12879_2015_1193_MOESM2_ESM.docx]

1. **Age-specific mean health outcomes per season in Canada**

|  | **TIV**  **n (95% CI)** | **QIV**  **n (95% CI)** | **Difference**  **n (95% CI)** | **% Difference**  **n (95% CI)** |
| --- | --- | --- | --- | --- |
| **0–4 years** | | | | |
| Cases | 266218 (235144; 302789) | 252960 (223195; 287226) | -13258 (-20646; -8264) | -5.0 (-7.6; -3.2) |
| GP visits | 121129 (106990; 137769) | 115097 (101554; 130688) | -6032 (-9394; -3760) | -5.0 (-7.6; -3.2) |
| ER visits | 6794 (6001; 7727) | 6456 (5696; 7330) | -338 (-527; -211) | -5.0 (-7.6; -3.2) |
| Hospitalizations | 3754 (3316; 4269) | 3567 (3147; 4050) | -187 (-291; -117) | -5.0 (-7.6; -3.2) |
| Deaths | 11 (9; 12) | 10 (9; 11) | -1 (-1; 0) | -5.0 (-7.6; -3.2) |
| **5–19 years** | | | | |
| Cases | 566688 (489747; 645471) | 542466 (465387; 618874) | -24221 (-38946; -14626) | -4.3 (-6.8; -2.6) |
| GP visits | 179920 (155495; 204930) | 172229 (147764; 196482) | -7691 (-12366; -4645) | -4.3 (-6.8; -2.6) |
| ER visits | 988 (848; 1131) | 948 (806; 1087) | -41 (-68; -24) | -4.1 (-6.8; -2.4) |
| Hospitalizations | 546 (469; 625) | 523 (445; 601) | -23 (-37; -13) | -4.1 (-6.8; -2.4) |
| Deaths | 10 (9; 12) | 10 (8; 11) | 0 (-1; 0) | -4.1 (-6.8; -2.4) |
| **20–49 years** | | | | |
| Cases | 1316489 (1136295; 1503404) | 1263216 (1081597; 1444672) | -53273 (-89103; -30831) | -4.0 (-6.7; -2.4) |
| GP visits | 412061 (355660; 470566) | 395387 (338540; 452182) | -16674 (-27889; -9650) | -4.0 (-6.7; -2.4) |
| ER visits | 10008 (8638; 11429) | 9603 (8222; 10982) | -405 (-677; -234) | -4.0 (-6.7; -2.4) |
| Hospitalizations | 5529 (4772; 6314) | 5306 (4543; 6068) | -224 (-374; -129) | -4.0 (-6.7; -2.4) |
| Deaths | 118 (102; 135) | 114 (97; 130) | -5 (-8; -3) | -4.0 (-6.7; -2.4) |
| **50–64 years** | | | | |
| Cases | 432127 (368561; 499095) | 412697 (347580; 477183) | -19430 (-33835; -9993) | -4.5 (-7.8; -2.3) |
| GP visits | 135256 (115360; 156217) | 129174 (108793; 149358) | -6082 (-10590; -3128) | -4.5 (-7.8; -2.3) |
| ER visits | 15095 (12875; 17435) | 14417 (12142; 16669) | -679 (-1182; -349) | -4.5 (-7.8; -2.3) |
| Hospitalizations | 8340 (7113; 9633) | 7965 (6708; 9210) | -375 (-653; -193) | -4.5 (-7.8; -2.3) |
| Deaths | 579 (494; 669) | 553 (466; 639) | -26 (-45; -13) | -4.5 (-7.8; -2.3) |
| **65–74 years** | | | | |
| Cases | 190464 (162214; 220556) | 177776 (150287; 206369) | -12688 (-22542; -6051) | -6.6 (-11.7; -3.2) |
| GP visits | 118088 (100572; 136745) | 110221 (93178; 127949) | -7866 (-13976; -3751) | -6.6 (-11.7; -3.2) |
| ER visits | 14514 (12361; 16807) | 13547 (11452; 15726) | -967 (-1718; -461) | -6.6 (-11.7; -3.2) |
| Hospitalizations | 8019 (6829; 9285) | 7484 (6327; 8688) | -534 (-949; -255) | -6.6 (-11.7; -3.2) |
| Deaths | 2228 (1898; 2581) | 2080 (1758; 2415) | -148 (-264; -71) | -6.6 (-11.7; -3.2) |
| **75–84 years** | | | | |
| Cases | 114966 (97973; 133823) | 105945 (89322; 123201) | -9021 (-16197; -4119) | -7.8 (-13.7; -3.6) |
| GP visits | 71279 (60743; 82970) | 65686 (55380; 76385) | -5593 (-10042; -2553) | -7.8 (-13.7; -3.6) |
| ER visits | 8761 (7466; 10197) | 8073 (6806; 9388) | -687 (-1234; -314) | -7.8 (-13.7; -3.6) |
| Hospitalizations | 4840 (4125; 5634) | 4460 (3760; 5187) | -380 (-682; -173) | -7.8 (-13.7; -3.6) |
| Deaths | 1345 (1146; 1566) | 1240 (1045; 1441) | -106 (-190; -48) | -7.8 (-13.7; -3.6) |
| **85–99 years** | | | | |
| Cases | 46508 (39434; 53898) | 42861 (36056; 49865) | -3647 (-6533; -1629) | -7.8 (-13.5; -3.6) |
| GP visits | 28835 (24449; 33417) | 26574 (22355; 30916) | -2261 (-4050; -1010) | 7.8 (-13.5; -3.6) |
| ER visits | 3544 (3005; 4107) | 3266 (2747; 3800) | -278 (-498; -124) | 7.8 (-13.5; -3.6) |
| Hospitalizations | 1958 (1660; 2269) | 1804 (1518; 2099) | -154 (-275; -69) | 7.8 (-13.5; -3.6) |
| Deaths | 544 (461; 631) | 501 (422; 583) | -43 (-76; -19) | 7.8 (-13.5; -3.6) |

Note: A negative value for the difference denotes outcomes prevented; GP, general practitioner; ER, emergency room; QALY, quality-adjusted life year; LY, life year; TIV, trivalent influenza vaccine; QIV, quadrivalent influenza vaccine
