# Supplementary material for: Cost-effectiveness evaluation of quadrivalent influenza vaccines for seasonal influenza prevention: a dynamic modeling study of Canada and the United Kingdom
Source: BMC Infect Dis. 2015 Oct 27;15:465. doi: 10.1186/s12879-015-1193-4 (PMC4623926; doi:10.1186/s12879-015-1193-4)
Supplement: Additional file 3: — Age-specific mean health outcomes per season in the UK. (DOCX 65 kb) [file 12879_2015_1193_MOESM3_ESM.docx]

1. **Age-specific mean health outcomes per season in the UK**
2. **Model outcomes for UK Scenario 1**

|  | **TIV/LAIV** | **QIV/QLAIV** | **Difference (TIV/LAIV vs QIV/QLAIV)** | **% Difference** |
| --- | --- | --- | --- | --- |
| **0–4 years** | | | | |
| Cases | 1,702,906 (1,484,363; 1,954,740) | 1,685,226 (1,467,326; 1,928,467) | -17,680 (-32,022; -8,517) | -1.0 (-1.9; -0.5) |
| GP visits | 264,121 (230,225; 303,180) | 261,379 (227,582; 299,105) | -2,742 (-4,967; -1,321) | -1.0 (-1.9; -0.5) |
| ER visits | 8,169 (7,120; 9,377) | 8,084 (7,039; 9,251) | -85 (-154; -41) | -1.0 (-1.9; -0.5) |
| Hospitalizations | 26,225 (22,859; 30,103) | 25,952 (22,597; 29,698) | -272 (-493; -131) | -1.0 (-1.9; -0.5) |
| Deaths | 0 (0; 0) | 0 (0; 0) | 0 (0; 0) | - |
| **5–19 years** | | | | |
| Cases | 891,782 (768,652; 1,026,512) | 881,824 (758,238; 1,015,506) | -9,959 (-17,169; -5,464) | -1.1 (-1.9; -0.6) |
| GP visits | 187,815 (161,816; 216,154) | 185,701 (159,576; 213,818) | -2,114 (-3,650; -1,154) | -1.1 (-1.9; -0.6) |
| ER visits | 5,809 (5,005; 6,685) | 5,743 (4,935; 6,613) | -65 (-113; -36) | -1.1 (-1.9; -0.6) |
| Hospitalizations | 11,946 (10,300; 13,755) | 11,813 (10,160; 13,607) | -132 (-228; -73) | -1.1 (-1.9; -0.6) |
| Deaths | 81 (70; 94) | 80 (69; 92) | -1 (-2; -1) | -1.2 (-2.0; -0.6) |
| **20–49 years** | | | | |
| Cases | 2,522,554 (2,171,957; 2,897,690) | 2,493,223 (2,137,056; 2,870,378) | -29,331 (-50,499; -16,015) | -1.2 (-2.0; -0.6) |
| GP visits | 711,108 (612,275; 816,859) | 702,840 (602,436; 809,160) | -8,269 (-14,236; -4,515) | -1.2 (-2.0; -0.6) |
| ER visits | 21,993 (18,936; 25,264) | 21,737 (18,632; 25,026) | -256 (-440; -140) | -1.2 (-2.0; -0.6) |
| Hospitalizations | 22,198 (19,113; 25,500) | 21,940 (18,806; 25,259) | -258 (-444; -141) | -1.2 (-2.0; -0.6) |
| Deaths | 757 (652; 869) | 748 (641; 861) | -9 (-15; -5) | -1.2 (-2.0; -0.6) |
| **50–64 years** | | | | |
| Cases | 690,541 (594,254;795,848) | 678,012 (578,488; 784,572) | -12,529 (-21,964; -6,501) | -1.8 (-3.1; -0.9) |
| GP visits | 194,664 (167,520; 224,349) | 191,132 (163,076; 221,171) | -3,532 (-6,192; -1,833) | -1.8 (-3.1; -0.9) |
| ER visits | 6,021 (5,181; 6,939) | 5,911 (5,044; 6,840) | -109 (-191; -57) | -1.8 (-3.1; -0.9) |
| Hospitalizations | 6,077 (5,229; 7,003) | 5,967 (5,091; 6,904) | -110 (-193; -57) | -1.8 (-3.1; -0.9) |
| Deaths | 483 (416; 557) | 475 (405; 549) | -9 (-15; -5) | -1.8 (-3.1; -0.9) |
| **65–74 years** | | | | |
| Cases | 216,259 (185,365; 250,205) | 206,211 (175,267; 238,834) | -10,048 (-18,452; -4,633) | -4.6 (-8.2; -2.2) |
| GP visits | 70,306 (60,262; 81,342) | 67,039 (56,979; 77,645) | -3,267 (-5,999; -1,506) | -4.6 (-8.2; -2.2) |
| ER visits | 2,174 (1,864; 2,516) | 2,073 (1,762; 2,401) | -101 (-186; -47) | -4.6 (-8.2; -2.2) |
| Hospitalizations | 3,114 (2,669; 3,603) | 2,969 (2,524; 3,439) | -145 (-266; -67) | -4.6 (-8.2; -2.2) |
| Deaths | 2,379 (2,039; 2,752) | 2,268 (1,928; 2,627) | -111 (-203; -51) | -4.6 (-8.2; -2.2) |
| **75–84 years** | | | | |
| Cases | 120,507 (103,357; 140,177) | 113,204 (95,818; 131,201) | -7,303 (-13,669; -3,325) | -6.0 (-10.6; -2.8) |
| GP visits | 39,177 (33,601; 45,572) | 36,802 (31,150; 42,654) | -2,374 (-4,444; -1,081) | -6.0 (-10.6; -2.8) |
| ER visits | 1,212 (1,039; 1,409) | 1,132 (963; 1,319) | -73 (-137; -33) | -6.0 (-10.6; -2.8) |
| Hospitalizations | 1,735 (1,488; 2,019) | 1,630 (1,380; 1,889) | -105 (-197; -48) | -6.0 (-10.6; -2.8) |
| Deaths | 1,326 (1,137; 1,542) | 1,245 (1,054; 1,443) | -80 (-150; -37) | -6.0 (-10.6; -2.8) |
| **85–99 years** | | | | |
| Cases | 30,923 (26,498; 35,973) | 29,019 (24,498; 33,179) | -1,905 (-3,556; -863) | -6.1 (-10.7; -2.8) |
| GP visits | 10,053 (8,615; 11,695) | 9,434 (7,964; 10,962) | -619 (-1,156; -280) | -6.1 (-10.7; -2.8) |
| ER visits | 311 (266; 362) | 292 (246; 339) | -19 (-36; -9) | -6.1 (-10.7; -2.8) |
| Hospitalizations | 445 (382; 518) | 418 (353; 486) | -27 (-51; -12) | -6.1 (-10.7; -2.8) |
| Deaths | 340 (291; 396) | 319 (269; 371) | -21 (-39; -9) | -6.1 (-10.7; -2.8) |

Note: A negative value for the difference denotes outcomes prevented; GP, general practitioner; ER, emergency room; QALY, quality-adjusted life year; LY, life year; TIV, trivalent influenza vaccine; QIV, quadrivalent influenza vaccine

1. **Model outcomes for UK Scenario 2**

|  | **TIV/LAIV** | **QIV/QLAIV** | **Difference (TIV/LAIV vs QIV/QLAIV)** | **% Difference** |
| --- | --- | --- | --- | --- |
| **0–4 years** | | | | |
| Cases | 1,487,156 (1,316,543; 1,692,445) | 1,466,488 (1,293,428; 1,665,800) | -20,668 (-36,970; -10,333) | -1.4 (-2.4; -0.7) |
| GP visits | 230,658 (204,196; 262,498) | 227,452 (200,611; 258,366) | -3,206 (-5,734; -1,603) | -1.4 (-2.4; -0.7) |
| ER visits | 7,134 (6,315; 8,119) | 7,035 (6,204; 7,991) | -99 (-117; -50) | -1.4 (-2.4; -0.7) |
| Hospitalizations | 22,902 (20,275; 26,064) | 22,584(19,919; 25,653) | -318(-569; -159) | -1.4 (-2.4; -0.7) |
| Deaths | 0 (0; 0) | 0 (0; 0) | 0 (0; 0) | - |
| **5–19 years** | | | | |
| Cases | 705,468 (611,754; 809,273) | 694,713 (600,921; 795,666) | -10,755 (-18,160; -6,132) | -1.5 (-2.5; -0.9) |
| GP visits | 149,126 (129,373; 171,049) | 146,842 (126,996; 168,133) | -2,284 (-3,853; -1,297) | -1.5 (-2.5; -0.9) |
| ER visits | 4,612 (4,001; 5,290) | 4,541 (3,928; 5,200) | -71 (-119; -40) | -1.5 (-2.5; -0.9) |
| Hospitalizations | 9,411 (8,157; 10,798) | 9,268 (8,016; 10,619) | -143 (-242; -82) | -1.5 (-2.5; -0.9) |
| Deaths | 66 (57; 76) | 65 (56; 74) | -1 (-2; -1) | -1.6 (-2.6; -0.9) |
| **20–49 years** | | | | |
| Cases | 2,206,393 (1,915,183; 2,524,286) | 2,172,917 (1,880,211; 2,483,294) | -33,476 (-57,074; -18,956) | -1.5 (-2.5; -0.9) |
| GP visits | 621,982 (539,890;711,596) | 612,545 (530,031; 70,041) | -9,437 (-16,089; -5,344) | -1.5 (-2.5; -0.9) |
| ER visits | 19,237 (16,698;22,008) | 18,945 (16,393; 21,651) | -292 (-498; -165) | -1.5 (-2.5; -0.9) |
| Hospitalizations | 19,416 (16,854;22,214) | 19,122 (16,546; 21,853) | -295 (-502; -167) | -1.5 (-2.5; -0.9) |
| Deaths | 662 (575;757) | 652 (564; 745) | -10 (-17; -6) | -1.5 (-2.5; -0.9) |
| **50–64 years** | | | | |
| Cases0 | 607,398 (526,939; 696,061) | 593,966 (514,378; 682,629) | -13,432 (-22,958; -7,212) | -2.2 (-3.6; -1.2) |
| GP visits | 171,226 (148,544; 196,220) | 167,439 (145,003; 192,433) | -3,787 (-6,472; -2,033) | -2.2 (-3.6; -1.2) |
| ER visits | 5,296 (4,594; 6,069) | 5,179 (4,485; 5,952) | -117 (-200; -63) | -2.2 (-3.6; -1.2) |
| Hospitalizations | 5,345 (4,637; 6,125) | 5,227 (4,527; 6,007) | -118 (-202; -63) | -2.2 (-3.6; -1.2) |
| Deaths | 425 (369; 487) | 416 (360; 478) | -9 (-16; -5) | -2.2 (-3.6; -1.2) |
| **65–74 years** | | | | |
| Cases | 192,275 (167,175; 220,650) | 182,114 (157,264; 209,022) | -10,162 (-17,816; -4,968) | -5.3 (-9.0; -2.7) |
| GP visits | 62,509 (54,349; 71,733) | 59,205 (51,127; 67,953) | -3,304 (-5,792; -1,615) | -5.3 (-9.0; -2.7) |
| ER visits | 1,933 (1,681; 2,219) | 1,831 (1,581; 2,102) | -102 (-179; -50) | -5.3 (-9.0; -2.7) |
| Hospitalizations | 2,769 (2,407; 3,177) | 2,622 (2,265; 3,010) | -146 (-257; -72) | -5.3 (-9.0; -2.7) |
| Deaths | 2,115 (1,839; 2,427) | 2,003 (1,730; 2,299) | -112 (-196; -55) | -5.3 (-9.0; -2.7) |
| **75–84 years** | | | | |
| Cases | 108,805 (94,421; 125,134) | 101,432 (87,293; 116,390) | -7,373 (-12,908; -3,558) | -6.8 (-11.5; -3.4) |
| GP visits | 35,373 (30,696; 40,681) | 32,976 (28,379; 37,838) | -2,397 (-4,196; -1,157) | -6.8 (-11.5; -3.4) |
| ER visits | 1,094 (949; 1,258) | 1,020 (878; 1,170) | -74 (-130; -36) | -6.8 (-11.5; -3.4) |
| Hospitalizations | 1,567 (1,360; 1,802) | 1,461 (1,257; 1,676) | -106 (-186; -51) | -6.8 (-11.5; -3.4) |
| Deaths | 1,197 (1,039; 1,376) | 1,116 (960; 1,280) | -81 (-142; -39) | -6.8 (-11.5; -3.4) |
| **85–99 years** | | | | |
| Cases | 27,936 (24,167; 32,196) | 26,015 (22,327; 29,983) | -1,922 (-3,408; -935) | -6.9 (-11.9; -3.4) |
| GP visits | 9,082 (7,857; 10,467) | 8,457 (7,258; 9,748) | -625 (-1,108; -304) | -6.9 (-11.9; -3.4) |
| ER visits | 281 (243; 324) | 262 (224; 301) | -19 (-34; -9) | -6.9 (-11.9; -3.4) |
| Hospitalizations | 402 (348; 464) | 375 (322; 432) | -28 (-49; -13) | -6.9 (-11.9; -3.4) |
| Deaths | 307 (266; 354) | 286 (246; 330) | -21 (-37; -10) | -6.9 (-11.9; -3.4) |

Note: A negative value for the difference denotes outcomes prevented; GP, general practitioner; ER, emergency room; QALY, quality-adjusted life-year; LY, life year; TIV, trivalent influenza vaccine; QIV, quadrivalent influenza vaccine; LAIV, live-attenuated influenza vaccine (trivalent); QLAIV, quadrivalent LAIV
